# A rapid tool for understanding how knowledge users engage with research findings in research-for-development contexts

**DOI:** 10.1186/s12961-026-01478-1

**Published:** 2026-04-11

**Authors:** Steven Lam, Vivian Hoffmann, Lilian Otoigo, Hung Nguyen-Viet

**Affiliations:** 1 International Livestock Research Institute, Naivasha Road, P.O. box 30709-00100, Nairobi, Kenya; 2 International Food and Policy Research Institute, Washington, United States of America; 3 Department of Economics and School of Public Policy and Administration, Carleton University, Ottawa, Canada; 4 International Livestock Research Institute, Hanoi, Vietnam

**Keywords:** One health, Research evaluation, Research impact, Knowledge translation, Africa, Asia

## Abstract

Promoting the use of research findings in development projects is essential but often overlooked during study design. Existing frameworks for research use tend to focus on clinical settings and offer questionable applicability to development contexts, which are typically nonlinear, dynamic and cross-sectoral. As a result, there remains a gap in tools that can capture how evidence is intended to be applied by diverse knowledge users in real-world development settings. To address this gap, and drawing on over a decade of experience implementing research-to-action strategies, our objective is to develop a simple research uptake and use tool to better understand and support the use of evidence in research-for-development. We piloted the tool immediately after or 1 month following dissemination workshops, engaging 206 participants across nine sessions in five countries – Kenya, Ethiopia, Bangladesh, Malawi, and Vietnam – to gather insights on which evidence was most relevant, how participants intended to apply it and why they valued it. Although conceptualized with a focus on agriculture and global health research, this framework is broadly applicable across the wider development sector in low- and middle-income countries.

**Supplementary Information:**

The online version contains supplementary material available at 10.1186/s12961-026-01478-1.

## Background

In development settings, researchers are expected not only to share evidence but also to do so in ways that contribute to better outcomes. This process – known as knowledge translation (KT) – involves synthesizing, disseminating and applying knowledge [1]. Evaluating which knowledge is taken up and how it is used is key to understanding the effectiveness of KT efforts. There is growing recognition of this need, with researchers and funders calling for greater attention to KT evaluation [2, 3]. For example, Canada’s International Development Research Centre, along with UK Research and Innovation, have commissioned work to assess the impact of KT funding [4, 5].

A wide range of tools are available to assess research uptake and use, with a scoping review identifying 23 such tools [6]. Notably, most were designed for clinical, public health or educational settings; none were specifically conceptualized with research-for-development (R4D) contexts in mind – situations where research is applied to address nonlinear, dynamic and cross-sectoral challenges such as poverty or food insecurity. Moreover, since these tools were primarily developed in high-income countries, their applicability in low-resource settings – where research uptake is often lower [7] – remains uncertain.

Given the increased priority in demonstrating KT impact, and the limited tools tailored for R4D contexts, there is a need for new assessment tools. As such, the goal of this study was to develop, test and report on a new tool called Research Uptake and Use Evaluation (RUUE). RUUE is grounded in the existing literature and draws on more than a decade of experience implementing research management strategies to support R4D in agricultural contexts [8].

### The KT framework

RUUE aims to understand what, how and why research is taken up and used by different actors in R4D settings. The design of RUUE was informed by the Research and Policy in Development (RAPID) framework, which identifies four interrelated domains that influence whether research evidence is used by knowledge users: (1) political context; (2) the quality and relevance of the evidence; (3) links between researchers and policy actors; and (4) external influences [9]. RUUE seeks to surface not only these four domains but also extend the dimensions to incorporate type of use and potential impact, which are important considerations for understanding how to further efforts to promote uptake and use.

Of note, the RAPID framework emphasizes research–policy linkages, with less focus on research–program linkages. However, in R4D contexts, programming plays a central role – encompassing activities such as piloting new interventions or adapting existing ones. These programming efforts can, in turn, influence policy decisions. Additionally, many KT frameworks are lengthy – sometimes containing up to 50 survey items, which, while comprehensive, can lead to survey fatigue in contexts where development actors are balancing multiple priorities.

Examples such as Level of Knowledge Use Survey (LOKUS) [10]; Seeking, Engaging with and Evaluating Research (SEER) [11]; and Knowledge Uptake and Utilization Tool (KUUT) [12] illustrate shared features: All view research use as a staged process and draw on established theories of knowledge uptake. LOKUS tracks progression from non-awareness to use; SEER measures policy-makers’ capacity, behaviours and intentions; and KUUT spans awareness through implementation and impact. Despite their different origins, these tools highlight the multidimensional nature of research utilization.

Drawing on selected core concepts from these frameworks, along with our field experience, we developed a simplified framework adapted to R4D contexts.

**Fig. 1:**
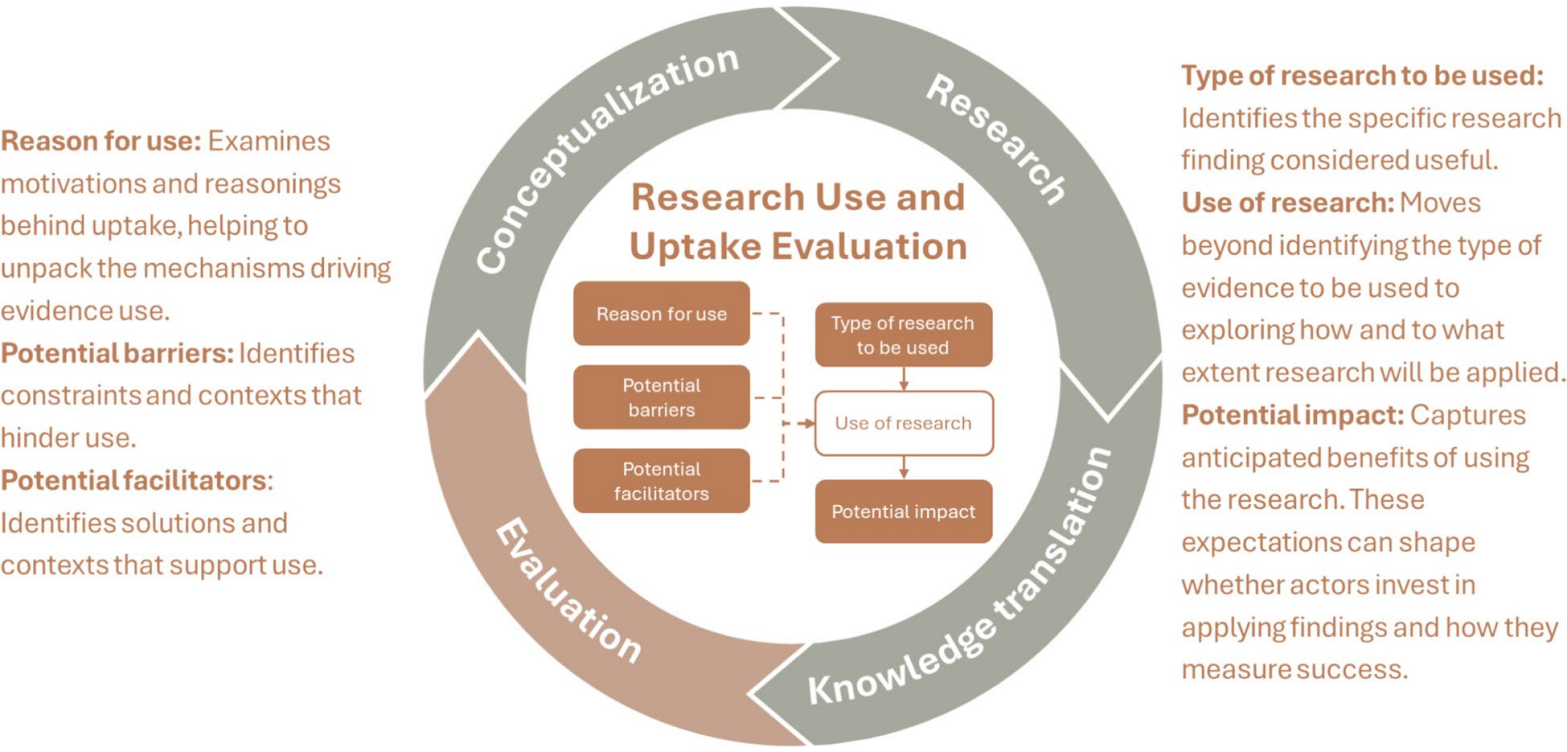
This visual illustrates how the items of the research uptake and use tool span across the research-to-action cycle, particularly evaluation. The circular representation demonstrates the dynamic nature of the process; evaluation may inform ongoing R4D efforts. Arrows within the circle depict how findings lead to application, influenced by reasonings, barriers and facilitators

The RUUE tool is a framework designed to assess how research evidence is adopted and applied in R4D contexts. While the decision to adopt evidence often occurs toward the end of the research-to-action cycle – during the evaluation phase – RUUE emphasizes that use should be deliberately considered throughout the stages of the research process, in line with R4D principles. The framework comprises six items for assessing research uptake and use (Fig. 1): type of research to be used, use of research, reasons for use, potential impact, potential barriers and potential facilitators.

### Case study of the CGIAR initiative on One Health

We tested the framework with the CGIAR One Health Initiative (OHI) (19 million US dollars; 2022–2024), which aimed to address health challenges in food systems. This initiative was guided by One Health, an approach that recognizes that the health of people, animals and the environment are intertwined, and, as such, advocates for addressing health risks through a multisectoral approach, involving experts from fields such as human health, veterinary medicine, food safety and environmental science [13].

The initiative was operationalized through five thematic work packages (WPs): (1) zoonoses; (2) food safety; (3) antimicrobial resistance (AMR); (4) water safety; and (5) governance. Each WP sought to generate solutions, develop capacity for their use and influence practice and policy. Of note, the five WPs reflected diverse starting points, with some building on a longer history of research engagement – such as zoonoses and food safety – while others, such as AMR, represented more recent areas of work. WPs operated in seven countries in Africa and Asia: Vietnam, India, Bangladesh, Ethiopia, Kenya, Uganda and Côte d’Ivoire.

CGIAR offers a strong case study for developing RUUE. As a global research partnership focused on transforming food systems, CGIAR carries out R4D. This dual focus makes CGIAR an ideal context to examine how evidence is used and to assess the impact of this use across both research and development domains. Evaluations of implementation processes and lessons learned from CGIAR OHI have been published elsewhere [14].

## Methods

### Research design

This study used a mixed-methods design to develop and pilot the RUUE tool. Tool development began with a rapid review of existing qualitative and quantitative instruments that assess knowledge use. The review focused specifically on tools measuring the use of research evidence, as distinct from tools assessing engagement with other knowledge resources such as websites or training sessions. Findings from the review informed the development of a survey organized around the six RUUE items (Table 1). The survey was then applied in results dissemination workshop settings, with responses analysed both qualitatively and quantitatively. A summary of selected tools, their key characteristics and how they compare with RUUE is provided in Additional File 1.

**Table 1:** Survey questions on research uptake and use along with demographic information

Survey questions	Demographic information
1. What finding from the research will you use?	Organization/occupation
2. How will you apply this research finding?	County/province
3. What are the main reasons for using this evidence in your work?	Country
4. Can you describe the potential impact of using this evidence?	Gender
5. What challenges do you anticipate when using this evidence, and in what situations do these challenges arise?	
6. What strategies do you think would help this research evidence be used, and in what situations will this work?	

### Study population and sampling

Results dissemination workshops were conducted between July and December 2024. All workshop attendees were invited to complete the survey, while interview participants were purposively selected to ensure a balance of respondent type and gender. Recognizing the diversity of knowledge users – including researchers, governmental officials and community members – RUUE also considers user demographics to better understand how different groups apply evidence and the distinct pathways through which it is used.

### Data collection and analysis

Surveys were primarily administered at the end of these sessions, except in Kenya, where follow-up interviews with respondents (*n* = 20) were conducted 1 month later. Participants were given approximately 45 min to complete the survey, with interviews lasting a similar duration. The open-ended questions were first analysed thematically to identify patterns or themes in the data [15]. We applied a deductive coding approach, coding text segments under broad themes that align with survey items. The lead author synthesized initial themes, which were then refined through discussions with the co-authors.

Of note, for the item “use of research”, we later quantified the responses to understand not only the type of use but also to what extent. We counted each coded segment and produced frequency summaries to show how often themes appeared. In addition, for the items “potential barriers” and “potential facilitators”, we wanted to avoid presenting a list of common-sense barriers and facilitators [16]. To do so, we explored underlying beliefs influencing research use practices by asking “Why do these exist, and in what context do they manifest?”

Ethics approval for this work was granted from International Livestock Research Institute’s Institutional Research Ethics Committee (ILRI-IREC2024-36).

## Results

### Overview of participants

The inclusion criteria included attendance at a dissemination workshop. Eligible participants were those who attended at least one full session and were affiliated with institutions involved in research or implementation activities relevant to the disseminated findings. A total of 206 participants took part in the study through nine workshops held across five countries: Kenya, Ethiopia, Bangladesh, Malawi and Vietnam. Participants represented a wide range of professional backgrounds and institutions. Men made up most of the participants overall, although gender balance varied by setting – ranging, for example, from mostly men in Ethiopia and Kenya, to more even representation in Vietnam. Most workshops included participants from government agencies at county, provincial and national levels (primarily ministries and departments of health, agriculture, environment and veterinary services) as well as researchers and non-governmental organizations (NGOs), and in some cases, private sector representatives and community members participating in interventions.

### Research findings: high intentions to use research evidence, focus on evidence of health risks versus interventions, and variation in evidence uptake by work package maturity

Most participants (88%) specified examples of research evidence they intend on using. In WP1 zoonoses workshops (Malawi and Vietnam), 34 of 42 participants (88%) cited an example. In WP2 food safety workshops (Ethiopia and Vietnam), all 73 participants identified evidence. Uptake was lower in WP3 AMR workshops (Bangladesh and Vietnam) – a comparatively newer One Health focus area – with 29 of 42 participants (69%) identifying evidence. WP4 water workshops (Ethiopia) had 8 of 9 participants (89%) cite evidence, while WP5 governance workshops (Kenya) again had all participants provide an example (20 of 20). The cross-cutting WP workshop (Vietnam) saw 17 of 20 participants (85%) provide examples.

Overall, the most frequently cited evidence concerned health risk analyses, followed by intervention studies outlining strategies to reduce those risks. For zoonoses, participants referred to evidence on pathogens and wildlife-related risk factors. Food safety evidence centred on hygiene standards, water quality monitoring, provision of equipment and slaughterhouse worker training. Environmental health examples included studies on water pollution, wastewater treatment and biodiversity loss. AMR evidence highlighted resistance in environmental settings.

### Application of findings: most commonly conceptual use, with less frequent application for awareness-raising, problem-solving, capacity-building or strategic purposes

**Fig. 2:**
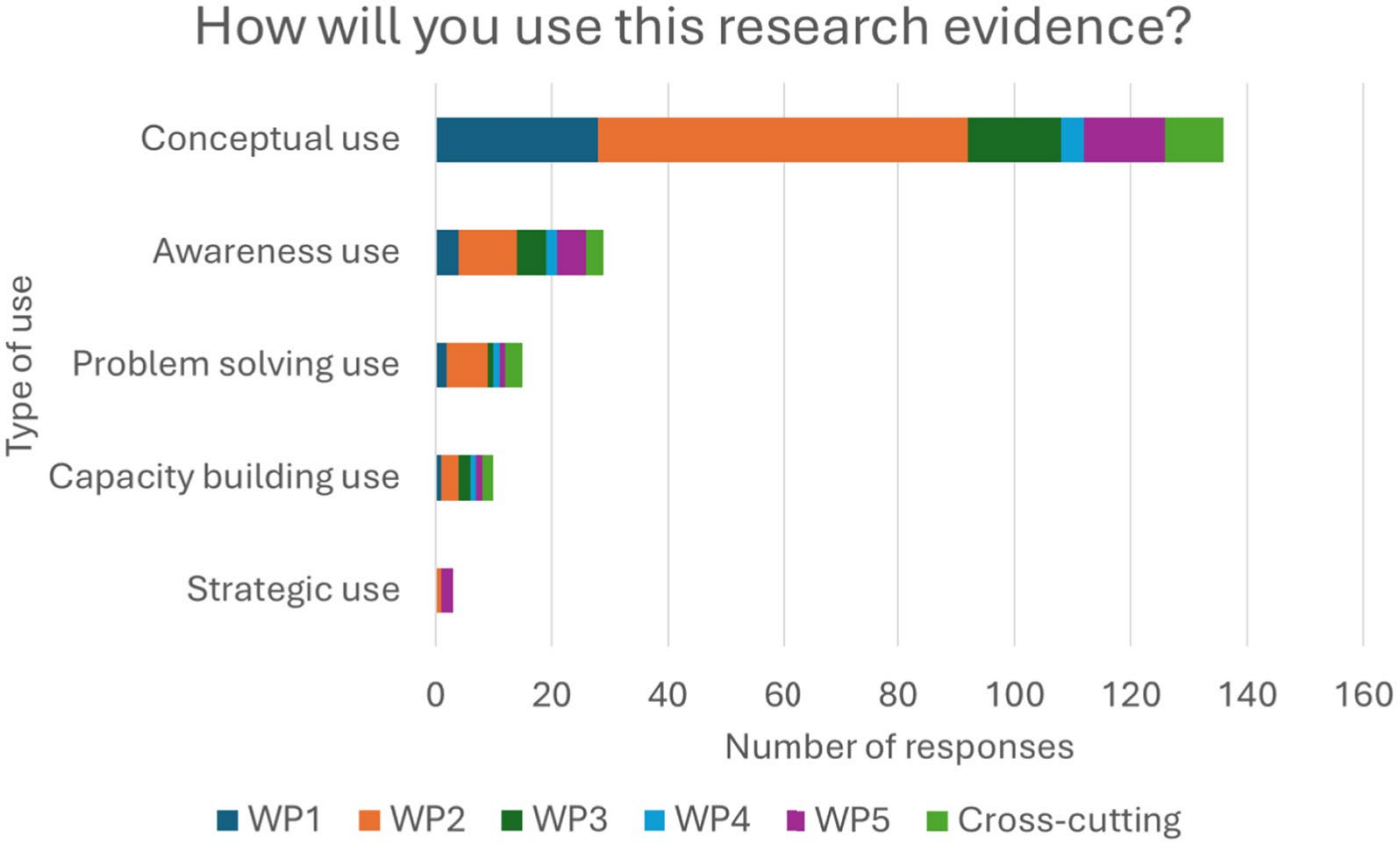
Extent of research use

From the open-ended responses, we categorized the different types of research uses as conceptual use, awareness use, problem-solving use, capacity-building use and strategic use (Table 2). Among the 193 responses, the most common use was conceptual (70%, *n* = 136), followed by awareness (15%, n = 29), problem-solving (8%, *n* = 15), capacity-building (5%, *n* = 10) and strategic (2%, *n* = 3) (Fig. 2). When disaggregated by WP, all WPs showed predominately conceptual use, suggesting that further translation support may be needed to move from understanding to action. Only WP2 and WP5 showed any strategic use, indicating a higher level of readiness among participants or clearer pathways for applying evidence in these areas.

**Table 2:** Type of research use

Construct	Definition	Example
Conceptual use	To enhance understanding of an issue without immediate application to decision-making	“The research results changed my perspective on how to use antibiotics in livestock farming”
Awareness use	To raise awareness among policy-makers, stakeholders or the general public	“I will use the evidence of zoonotic disease risks to create communication materials to help livestock farmers to prevent spread”
Problem-solving use	To directly inform decision-making to address a specific challenge	“Use One Health interventions to inform appropriate measures to improve food safety in national monitoring programs”
Capacity-building use	To educate their colleagues and networks on a particular issue	“I will practice and share the information with colleagues”
Strategic use	To justify pre-existing actions or explain a particular position	“We were aware of this issue… with this evidence we asked the county to employ more meat inspectors so we can take care of this shortage”

Defined as using research to enhance understanding of an issue without immediate application to decision-making, conceptual use was the most common form of uptake. Examples of future use of evidence included strengthening surveillance systems, deepening understanding of wildlife-related risks and informing thinking about biodiversity loss and climate resilience.

Awareness use refers to applying research to raise awareness among policy-makers, stakeholders or the public. For example, participants reported drawing on AMR evidence to communicate the importance of responsible antibiotic use in livestock and aquaculture. Participants of food safety and water workshops highlighted raising awareness about hygiene, foodborne risks and pollution among community members and decision-makers.

Problem-solving use involves applying research directly to inform decisions that addresses a challenge. Participants cited using food safety evidence in the future to develop monitoring tools, case studies and training materials, while others mentioned applying zoonoses research to improve wildlife handling. Water workshops also provided examples, such as informing existing antimicrobial stewardship and pollution-monitoring programs.

Capacity-building use focuses on educating colleagues and networks. Food safety workshops emphasized embedding evidence into current staff training modules, while governance workshops emphasized applying findings to strengthen existing food hygiene inspection protocols.

Strategic use is defined as applying research to justify pre-existing actions or positions. Though less common, participants noted using zoonoses and governance evidence to back calls for stronger biosecurity standards and licensing requirements. In some cases, research will also support broader initiatives such as Nature4Health, a global initiative working nationally to prevent pandemics by strengthening the environmental aspects of One Health.

### Reasons for use: filling information gaps, aligning with personal needs and supporting policy priorities

Many emphasized that the research findings addressed unmet information needs, offering solutions that were practical. Reflecting on food safety in slaughterhouses, one county director of veterinary services in Kenya observed, “The findings made us realize that there are small solutions to big problems that we can implement on our own”. Others highlighted the timeliness of the work; in this same context above, the food safety intervention concluded with results shared within just a month. The findings were seen as credible, having been generated through rigorous methods.

Notably, the relevance to their daily lives – as agri-food workers, consumers or public health officials – was a strong motivator. The evidence was also valued for its contribution to institutional mandates and local and national priorities. Respondents noted that using the evidence will help to improve practices and reduce health risks.

### Expected impact: better health, economies and policy decisions

Respondents most commonly highlighted that behaviour changes among farmers and food workers promoted by the initiative could reduce contamination, improve hygiene in slaughterhouses and markets, and lower rates of foodborne and zoonotic diseases. Many also noted potential economic benefits, such as increased consumer trust, more efficient livestock production and opportunities for market growth.

At the policy level, the evidence was viewed as a resource for developing regulations, guiding resource allocation and supporting evidence-based decision-making. Several respondents emphasized its value in addressing data gaps, informing future research and setting up interdisciplinary collaborations. Environmental benefits were also briefly cited, including reduced waste and more sustainable farming and aquaculture practices.

Reflecting on a dissemination workshop in Vietnam that shared results from all work packages, a representative from the Department of Animal Health at provincial level remarked that the evidence will help “minimize the impact of animal and human diseases, check food safety and hygiene levels in slaughterhouses and markets, and monitor the use of antibiotics in animal husbandry”.

### Potential barriers: research remaining in academic spaces, structural challenges and competing priorities

A key barrier to applying the evidence was that research findings often remain confined to academic spaces. While participants valued the dissemination workshops, they emphasized the need for results to be translated into accessible formats and broadly shared with communities. Structural and institutional barriers, such as weak coordination among One Health sectors (e.g. health, agriculture and environment ministries) and underfunded implementation mechanisms further limit the promotion and use of evidence across sectors and within communities.

Slow policy adoption processes also hinder change, particularly when urgency is low, and other priorities dominate – common in agricultural R4D contexts where productivity often takes precedence over human, animal or environmental health. Reflecting on these systemic challenges in the context of water safety, a representative from the Addis Ababa City Environmental Protection Agency explained, “We observe various problems in the city, including a weak economy, which take priority. Yet data also show significant environmental hygiene issues, with pollution affecting the environment, water, and air”.

### Potential facilitators: translating evidence beyond publications, collaborating with the private sector and leveraging existing One Health platforms

Participants emphasized that research should go beyond publications and dissemination workshops to actively reach communities through policy briefs, training sessions and behaviour change campaigns. Highlighting the importance of translating evidence for local audiences, a representative from Conservation Research Africa in Malawi noted, “We need to do more research to bring evidence to the local people that co-existence (of humans and wildlife) is possible”.

Broad stakeholder engagement – including the private sector – was viewed as essential for adoption and sustainability, though some cautioned that private sector interests may sometimes conflict with public goals. Leveraging existing One Health platforms and networks at national, subnational and county levels was highlighted as a way to expand reach. Participants also underscored the need to align research with policy priorities and institutional mandates to ensure relevance, and to use participatory approaches to enhance practicality. Several participants acknowledged that promoting research use is challenging and often requires researchers to test and adapt different approaches to find what works best.

## Discussion

This study developed and tested the RUUE tool to assess how evidence generated in agricultural R4D contexts is taken up and used. By applying RUUE to the CGIAR One Health Initiative, we were able to explore what evidence was most salient to users, how it was intended to be applied and why actors across different sectors valued the research.

Health risk analyses (e.g. of wildlife value chains, of foodborne diseases, of antimicrobial use and of waterborne pathogens) were the most frequently cited type of evidence. Their predominantly conceptual use reflects the early stage of the WPs, as the OHI – implemented only for 3 years – had prioritized generating risk-based evidence to inform interventions. As implementation research expands, other types of evidence uses are expected, marking a shift from learning to application. Signs of this shift are already visible; for instance, evidence discussed in the WP5 workshop centred on findings from a slaughterhouse hygiene intervention study with respondents mentioning all types of evidence use. Future applications of RUUE could track whether and how this transition unfolds.

Existing literature on knowledge use revealed three distinct levels of knowledge use: conceptual, problem solving and symbolic (i.e. strategic) [17]. By framing survey questions as open-ended and coding responses thematically, we were able to capture additional forms of evidence use – such as awareness and capacity-building – an important feature in dynamic R4D settings.

The reasons for planned evidence use are consistent with the RAPID framework [9], showing that evidence is valued when it is credible and actionable. This study adds that relevance to lived realities was also a strong driver of uptake. We further identified barriers and facilitators to research use, and found it useful to ask “In what context do these occur?” to move beyond common-sense responses as well as link the findings to the actual situations that knowledge users are facing.

For R4D actors seeking practical ways to bridge the gap between evidence generation and use, the RUUE tool offers a promising approach. Integrating it at the end of a dissemination workshop required only 45 min yet generated valuable, targeted insights from knowledge users on how to enhance research impact. The instance where follow-up interviews were conducted a month later in Kenya, however, provided much deeper insights – though they required more time and resources to analyse. We encourage R4D actors to reflect on their own experiences and contexts when selecting the most appropriate approach.

R4D actors may also consider using the tool in combination with other research impact assessments to create a fuller picture of research uptake and use [18]. RUUE captures types of use that traditional metrics such as bibliometrics often miss [19], offering a proxy for real-world application and impact – particularly among nontraditional stakeholders who may not formally cite research outputs. It is important, however, to recognize the challenges of assessing research impact, including time lags between research and societal benefits, the nonlinear pathways from evidence to impact, and the diverse forms of impact and ways they can be measured [20]. These factors should be kept in mind when interpreting findings from RUUE.

The development of RUUE carries important theoretical, practice and policy implications. Theoretically, RUUE advances existing knowledge-use frameworks by demonstrating that evidence use in dynamic, multisectoral settings extends beyond the classic conceptual–instrumental–symbolic typology [17] to include additional forms such as awareness and capacity-building. From a practice perspective, the tool offers R4D initiatives a quick way to generate feedback from knowledge users; by complementing traditional impact metrics, RUUE helps teams to better track progress towards impact, especially among actors who engage with research but may not formally cite it. At the policy level, RUUE offers funders a way to assess early, realistic research contributions to policy processes, informing reporting requirements and expectations around impact. If adopted, it could help reduce pressures researchers face to demonstrate premature or linear policy change.

This study has limitations. First, it focused on intended rather than actual use of evidence. While intentions captured immediately after results dissemination offer timely insights, they may not reflect what ultimately happens in practice. Follow-up would be necessary to assess whether and how intentions translated into action. Second, owing to the self-administered implementation of surveys, demographic information and sector of employment were often left blank. We found that interviews were more effective in capturing these details, enabling better disaggregation of responses by knowledge-user type, though they required more time. One practical alternative may be to collect such data in small groups during workshops, which could balance depth with efficiency. Despite these limitations, this tool offers a promising way to present intended research use qualitatively and quantitatively. Furthermore, the tool’s application across various R4D domains (e.g. zoonoses, food safety and AMR) demonstrates its potential for use across sectors. Applying the tool in additional sectors would further validate and strengthen its generalizability.

## Conclusions

This study piloted and reflected on the use of the RUUE tool to rapidly capture how research is intended to be used in R4D contexts. Our experiences offer practical insights for research and funding organizations seeking quick yet meaningful ways to understand intended research use, a proxy indicator for future societal impact. Applying the tool to a multisectoral case study on agriculture and health across five countries in Africa and Asia reinforced a key reminder: Research does not end with publication or dissemination workshops – it extends to the communities and stakeholders who ultimately apply and benefit from the evidence.

## Supplementary Information

Additional file 1.

## Data Availability

Data sharing is not applicable to this article as no datasets were generated or analysed during the current study.
