# Supplementary material for: A rapid tool for understanding how knowledge users engage with research findings in research-for-development contexts
Source: Health Res Policy Syst. 2026 Apr 11;24:43. doi: 10.1186/s12961-026-01478-1 (PMC13185264; doi:10.1186/s12961-026-01478-1)
Supplement: Supplementary file 1 — Additional file 1. [file 12961_2026_1478_MOESM1_ESM.docx]

**Additional file 1. Existing measures of research uptake and use**

***Analysis of existing self-report measures prior to the development of RUUE***

To establish the need for the RUUE tool, we first conducted a rapid review of existing qualitative and quantitative instruments that assess the use of knowledge. Our focus was specifically on tools that measure the use of research evidence – as distinct from tools that assess engagement with other types of knowledge resources such as websites or training sessions. Examples of tools are provided in **Table S1**.

Most of the tools reviewed were rooted in staged models of research utilization, such as those drawing on Levels of Use, or Stages of Innovation. These models typically conceptualize research use as progressing through stages – from awareness and consideration to application and impact. Most of these tools originated in health research contexts, where evidence often informs decision-making in relatively structured systems, such as healthcare delivery.

In contrast, research for development (R4D) operates in more complex, politically dynamic environments. In these settings, the pathway from research to policy and practice is often nonlinear and iterative, shaped by diverse actors and political conditions ^1^. As such, R4D requires conceptual frameworks that account for these contextual elements.

Furthermore, many existing tools were lengthy – often comprising 40 to 50 survey items – which could create survey fatigue in contexts where community members and other decision-makers face competing priorities. Furthermore, while some instruments addressed barriers and enablers to research use – which account for complexity of development programming – their reliance on pre-defined response categories limited exploration of contextual factors that are harder to put in a box, such as power asymmetries or institutional norms.

**Table S1.** Description and assessment of tools covering constructs measured by Research Uptake and Use Evaluation (RUUE).

|  | Level of Knowledge Use Survey (LOKUS) ^2^ | Seeking, Engaging with and Evaluating Research (SEER) ^3^ | Knowledge Uptake and Utilization Tool (KUUT) ^4^ |
| --- | --- | --- | --- |
| Description |  |  |  |
| Purpose | To measure knowledge use of stakeholders | To measure individual capacity to engage with and use research | To measure outcomes of knowledge exchange |
| About the tool | 47 items distributed under four levels of use: 1) non-awareness, 2) awareness, 3) interest, and 4) use | 50 questions using Likert or binary scales to assess individual policymakers' capacity to use research/evidence, research engagement actions, and actual research use | 44-item questionnaire comprised of 10 domains which capture initial awareness and use of knowledge, to implementation and impact |
| Framework that informed the tool | LOKUS is structured along the Stages of Innovation Adoption ^5^, while its item generation protocol was guided by the Levels of Use Scale ^6^ | The measure of intention  to use research based on the theory of planned behaviour ^7^ | KUUT was informed by Level of Use Scale ^6^, as well as Seven Stages of Knowledge Utilization ^8^ |
| Context of tool development | Developed in the context of assessing knowledge communication strategies in rehabilitation technology | Developed in the context of assessing research use by health policymakers | Developed in the context of assessing knowledge exchange activities in health |
| Coverage of domains from RUUE | | | |
| Conceptual use | Not covered | **Covered** (under conceptual use) | **Covered** (under cognition construct) |
| Awareness use | **Covered** (under awareness use) | Not covered | Not covered |
| Problem solving use | Not covered | **Covered** (under instrumental use) | **Covered** (under adoption construct) |
| Capacity building use | Not covered | Not covered | **Covered** (under discussion construct) |
| Strategic use | Not covered | **Covered** (under tactical use) | Not covered |

**References**

1. Apgar, M., Snijder, M., Higdon, G. L. & Szabo, S. Evaluating Research for Development: Innovation to Navigate Complexity. *European Journal of Development Research* (2023) doi:10.1057/s41287-023-00577-x.

2. Lane, J. P., Stone, V. I., Nobrega, A. & Tomita, M. Level of Knowledge Use Survey (LOKUS): A validated instrument for tracking knowledge uptake and use. in *Studies in Health Technology and Informatics* (2015). doi:10.3233/978-1-61499-566-1-106.

3. Brennan, S. E. *et al.* Development and validation of SEER (Seeking, Engaging with and Evaluating Research): A measure of policymakers’ capacity to engage with and use research. *Heal. Res. Policy Syst.* (2017) doi:10.1186/s12961-016-0162-8.

4. Skinner, K. Developing a tool to measure knowledge exchange outcomes. *Can. J. Progr. Eval.* (2007).

5. Rogers, E. M. *Diffusion of Innovations*. (Univesity of Illinois, 1983).

6. Hall, G. E., Loucks, S. F., Rutherford, W. L. & Newlove, B. W. Levels of Use of the Innovation: A Framework for Analyzing Innovation Adoption. *J. Teach. Educ.* (1975) doi:10.1177/002248717502600114.

7. Boyko, J. A., Lavis, J. N., Dobbins, M. & Souza, N. M. Reliability of a tool for measuring theory of planned behaviour constructs for use in evaluating research use in policymaking. *Heal. Res. Policy Syst.* (2011) doi:10.1186/1478-4505-9-29.

8. Knott, J. & Wildavsky, A. If Dissemination Is the Solution, What Is the Problem? *Knowledge* (1980) doi:10.1177/107554708000100404.
